# Making sense of cardiomyopathy caused by deletion of acetyl-CoA carboxylase 1 and 2

**DOI:** 10.1172/JCI209270

**Published:** 2026-09-01

**Authors:** Rong Tian, E. Douglas Lewandowski

**Affiliations:** 1 Mitochondria and Metabolism Center, University of Washington, Seattle, Washington, USA.; 2 Department of Internal Medicine, The Ohio State University College of Medicine, Columbus, Ohio, USA.

**Keywords:** Cardiology, Metabolism, Fatty acid oxidation, Heart failure

**To the Editor:** Kim et al. report deletion of acetyl-CoA carboxylase (ACC) 1 and 2 in cardiomyocytes (dHKO) causes cardiomyopathy (1). They attribute the pathogenesis to unrestrained fatty acid oxidation (FAO) leading to the loss of cardiolipin and mitochondrial dysfunction. The two ACC isoforms are encoded by separate genes and have distinct functions (2, 3). ACC1 regulates lipid biosynthesis and acyl chain elongation, by producing malonyl-CoA in the cytosol. ACC2, on the other hand, is localized on mitochondria. There, ACC2 regulates FAO by producing malonyl-CoA, which inhibits carnitine palmitoyltransferase I, thus controlling entry of long chain fatty acids into mitochondria. Deletion of the two isoforms is expected to affect both lipid biosynthesis and oxidation, but evidence supporting the conclusion of the Kim et al. study is not convincing.

First, the increase of FAO observed in the dHKO is modest at approximately 15%. Compared with an earlier study (4) that deleted ACC2 in cardiomyocytes using the same gene-targeting strategy, FAO increased less in dHKO than in the single-gene deletion in the ACC2 HKO model (Table 1). Additionally, the single ACC2 HKO did not develop mitochondrial dysfunction or cardiomyopathy when observed for up to a year (Table 1). Kim et al. posit that deleting both ACC1/2 would further reduce malonyl-CoA to cause a greater increase of FAO. However, in the absence of data on FAO in ACC1 or ACC2 HKO hearts, it is not possible to ascertain the additive effects on FAO. Additionally, malonyl-CoA produced by ACC1 and by ACC2 are in two distinct compartments, and literature indicates they do not have redundant functions (2, 3).

The dHKO hearts developed phospholipid deficiency, with selective reduction of linolenyl phospholipids and tetralinoleoyl cardiolipin. This finding was attributed to an “excessive FAO” purported to exhaust linoleic acid supply, but direct evidence was lacking to support that high FAO selectively exhausts linoleic acid. Furthermore, dietary supplementation of linoleic acid did not rescue the dHKO phenotype (Supplemental Figure 6, D and E, in ref. 4). Similarly, suppressing FAO with etomoxir in adult dHKO did not improve but rather trended toward worsened cardiac function (Supplemental Figure 5 in ref. 4) These findings, collectively, call into question whether deprivation of fatty acids or specifically linoleic acid by high FAO is the cause of cardiomyopathy in dHKO hearts.

ACC1 catalyzes the first and rate-limiting step in fatty acid biosynthesis and fatty acid chain elongation. Deleting ACC1 was recently shown to alter the phospholipidome in platelets, suggesting an important role of ACC1 in sustaining lipidome homeostasis (5). Yet, its role in cardiomyocyte lipidome homeostasis, which is distinct from ACC2 and critical for mitochondrial membrane maintenance, was not examined by Kim et al. ACC1 plays a prominent role during development, and global ACC1-null mice die during development (2). The present study deleted cardiomyocyte ACC1/2 around birth using an *Myh6*-Cre, and the phenotype could be rescued only in young mice right after weaning, consistent with impaired maturation of dHKO hearts. The double deletion of ACC1/2 likely affects both fatty acid biosynthesis and oxidation in a critical window of postnatal mitochondrial maturation. Attributing cardiomyopathy to heightened FAO alone may be missing an opportunity to define the role of ACC1 in mitochondrial integrity and development.

Kim et al. concluded that “unrestrained fatty acid oxidation” directly compromised cardiolipin quality as a “trigger” of heart failure in their model. However, given the distinct and nonredundant functions of ACC1 and ACC2, these data are challenging to interpret in the absence of similar measures performed on each individual gene deletion and call into question the relevance of the double ACC1/2 cardiac deletion for normal and abnormal cardiac metabolism and in the setting of heart failure.

## Figures and Tables

**Table 1:**
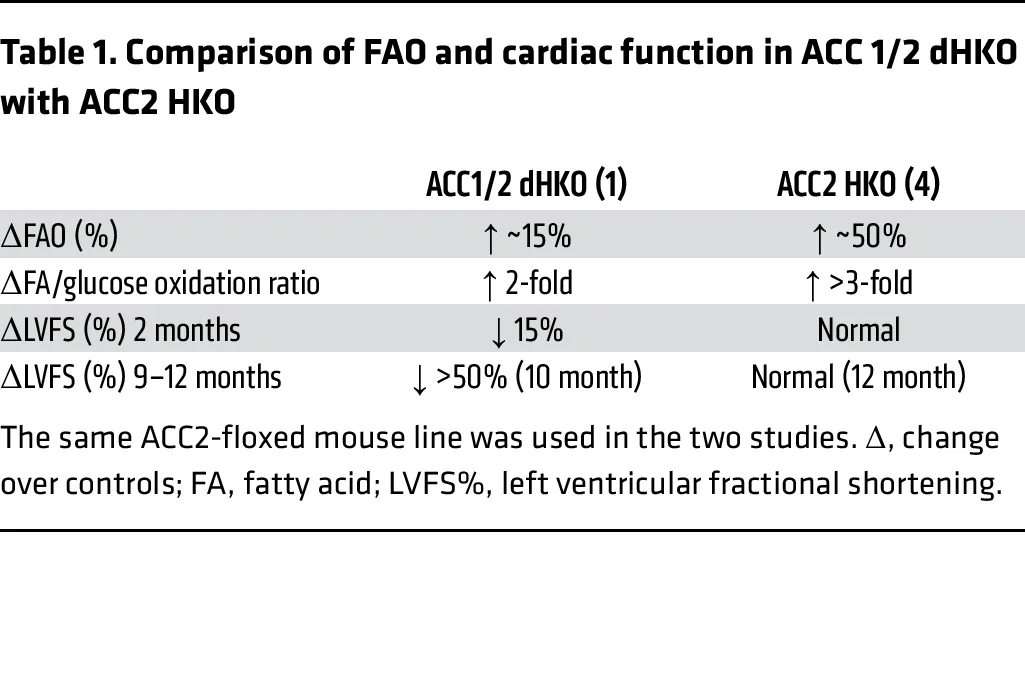
Comparison of FAO and cardiac function in ACC 1/2 dHKO with ACC2 HKO

## Version 1 09/01/2026

Electronic publication
